# Transcriptome Analysis of Long Noncoding RNAs in Toll-Like Receptor 3-Activated Mesenchymal Stem Cells

**DOI:** 10.1155/2016/6205485

**Published:** 2015-11-23

**Authors:** Shihua Wang, Xiaoxia Li, Robert Chunhua Zhao

**Affiliations:** Center of Excellence in Tissue Engineering, Institute of Basic Medical Sciences and Peking Union Medical College Hospital, School of Basic Medicine, Peking Union Medical College, Chinese Academy of Medical Sciences, Beijing 100005, China

## Abstract

Mesenchymal stem cells (MSCs) possess great immunomodulatory capacity which lays the foundation for their therapeutic effects in a variety of diseases. Recently, toll-like receptors (TLR) have been shown to modulate MSC functions; however, the underlying molecular mechanisms are poorly understood. Emerging evidence suggests that long noncoding RNAs (lncRNAs) are an important class of regulators involved in a wide range of biological processes. To explore the potential involvement of lncRNAs in TLR stimulated MSCs, we performed a comprehensive lncRNA and mRNA profiling through microarray. 10.2% of lncRNAs (1733 out of 16967) and 15.1% of mRNA transcripts (1760 out of 11632) were significantly differentially expressed (absolute fold-change ≥5 , *P* value ≤0.05) in TLR3 stimulated MSCs. Furthermore, we characterized the differentially expressed lncRNAs through their classes and length distribution and correlated them with differentially expressed mRNA. Here, we are the first to determine genome-wide lncRNAs expression patterns in TLR3 stimulated MSCs by microarray and this work could provide a comprehensive framework of the transcriptome landscapes of TLR3 stimulated MSCs.

## 1. Introduction

Mesenchymal stem cells (MSCs) are a type of adult stem cells with the capacity to generate a wide range of cells such as adipocytes, osteoblasts, chondrocytes, and myocytes [1]. Being originally identified from the bone marrow, MSCs now can be isolated from many other tissues including adipose tissue, placenta, and umbilical cord. MSCs also possess potent immunoregulatory abilities through interactions with both the adaptive and the innate immune cells. The immunomodulatory effect of MSCs is mediated by the secretion of soluble factors and/or by cell-cell contact-dependent regulation. These features make MSCs an interesting cell resource for tissue regeneration and cellular therapy.

Recently, the findings of functional toll-like receptors (TLRs) expression on MSC implicate these receptors in modulating MSCs functions [2]. TLRs are pattern recognition receptors involved in the recognition of pathogen-associated molecular patterns (PAMP) by immune cells, initiating both primary and adaptive immune responses [3]. To date, thirteen mammalian TLR analogs have been identified (10 in humans and 13 in mice). Beside their important roles in immunity, TLRs have also been recognized as regulators of stem cells functions, including cell growth, differentiation, and survival. For instance, De Luca et al. showed that TLR1/2 signaling instructed commitment of human hematopoietic stem cells to a myeloid cell fate [4]. Qi et al. demonstrated that both TLR3 and TLR4 promoted differentiation of bone marrow MSCs to osteoblasts [5].

In the field of immunology, recent publications have shown widespread changes in the expression of lncRNAs during the activation of the innate immune response and T cell development, differentiation, and activation [6]. It is well known that TLRs and their specific stimulus activate MyD88-dependent or -independent downstream signaling pathways. However, whether epigenetic regulators such as long noncoding RNA are involved in this process remains unclear. In this study, we determined the TLR expression profile of adipose tissue derived MSCs (AD-MSCs) and the consequences of TLR3 ligation in terms of cytokine secretion by these cells. To explore the potential involvement of lncRNAs in TLR stimulated MSCs, we performed comprehensive lncRNA and mRNA profiling through microarray. 10.2% of lncRNAs (1733 out of 16967) and 15.1% of mRNA transcripts (1760 out of 11632) were significantly differentially expressed (absolute fold-change ≥5, *P* value ≤ 0.05) in TLR3 stimulated MSCs. Furthermore, we characterized the differentially expressed lncRNAs through their classes and length distribution and correlated them with differentially expressed mRNA. To the best of our knowledge, this is the first study that links TLR3 with lncRNAs in MSCs.

## 2. Materials and Methods

### 2.1. Isolation and Culture of AD-MSCs

Adipose tissues were obtained from patients undergoing tumescent liposuction according to procedures approved by the Ethics Committee at the Chinese Academy of Medical Sciences and Peking Union Medical College. AD-MSCs were isolated and culture-expanded as previously reported [1, 7]. Passage 3 cells were used for the experiments.

### 2.2. Adipogenic and Osteogenic Differentiation of AD-MSCs

Adipogenic differentiation was induced in high glucose of Dulbecco's Modified Eagle's Medium (H-DMEM) supplemented with 10% FBS, 1 *μ*M dexamethasone, 0.5 mM 3-isobutyl-1-methylxanthine, and 5 *μ*g/mL 0.1 mM l-ascorbic acid. After being cultured in adipocyte induction medium for 4 days, medium was replaced with adipocyte maintaining medium, H-DMEM, with 10% FBS. For osteogenic differentiation, AD-MSCs were cultured in H-DMEM containing 10% FBS, 10 mM *β*-glycerophosphate, 50 *μ*mM l-ascorbic acid, and 0.01 *μ*M dexamethasone for 14 days.

### 2.3. ALP and Oil Red O Staining

The procedure of ALP staining was performed according to the manufacturer's instructions of ALP staining kit (Institute of Hematology and Blood Diseases Hospital, Chinese Academy of Medical Sciences). For oil red O staining, cells were washed twice with PBS and fixed with 10% formalin for 10 min at room temperature. After fixation, cells were stained with filtered oil red O solution (stock solution: 3 mg/mL in isopropanol, working solution: 60% oil red O stock solution and 40% distilled water) for 1 h at room temperature. After staining, cells were washed with water to remove unbound dye, visualized by light microscopy, and photographed.

### 2.4. Real-Time Polymerase Chain Reaction Analysis

Total RNA was extracted with TRIzol (Invitrogen), and cDNA was prepared. Real-time polymerase chain reaction (PCR) was amplified in triplicates according to manufacturer's procedures (TaKaRa). Relative expression of mRNA was evaluated by 2^−ΔΔCt^ method and normalized to the expression of GAPDH.

### 2.5. Cytokines Production

AD-MSCs were seeded in 12-well plate in culture medium with or without Poly I:C. Supernatants were collected after 24 h and concentrations of IL-1*β*, IL-4, IL-6, IL-8, IL-10, IL-12, TNF-*α*, TGF*β*, and IFN-*γ* were determined by enzyme-linked immunosorbent assay (ELISA) (R&D system) according to the manufacturer's instructions.

### 2.6. Microarray and Data Analysis

Arraystar Human LncRNA Microarray V3.0 is designed for the global profiling of human LncRNAs and protein-coding transcripts, which is updated from the previous microarray V2.0. About 30,586 LncRNAs and 26,109 coding transcripts can be detected by our third-generation LncRNA microarray. The LncRNAs are carefully constructed using the most highly respected public transcriptome databases (Refseq, UCSC knowngenes, Gencode, etc.), as well as landmark publications. Each transcript is represented by a specific exon or splice junction probe which can identify individual transcript accurately. Total RNA was extracted and examined for quality control before array. The OD260/OD280 ratios were approximately 2.0, and the OD260/OD230 ratios were more than 1.8 (Supplementary Table 1 in Supplementary Material available online at http://dx.doi.org/10.1155/2016/6205485). Positive probes for housekeeping genes and negative probes are also printed onto the array for hybridization quality control. (1) RNA labeling and array hybridization are as follows: sample labeling and array hybridization were performed according to the Agilent One-Color Microarray-Based Gene Expression Analysis protocol (Agilent Technology) with minor modifications. Briefly, mRNA was purified from total RNA after removal of rRNA (mRNA-ONLY Eukaryotic mRNA Isolation Kit, Epicentre). Then, each sample was amplified and transcribed into fluorescent cRNA along the entire length of the transcripts without 3′ bias utilizing a random priming method (Arraystar Flash RNA Labeling Kit, Arraystar). The labeled cRNAs were purified by RNeasy Mini Kit (Qiagen). The concentration and specific activity of the labeled cRNAs (pmol Cy3/*μ*g cRNA) were measured by NanoDrop ND-1000. 1 *μ*g of each labeled cRNA was fragmented by adding 5 *μ*L 10× blocking agent and 1 *μ*L of 25× fragmentation buffer and then the mixture was heated at 60°C for 30 min; finally, 25 *μ*L 2× GE hybridization buffer was added to dilute the labeled cRNA. 50 *μ*L of hybridization solution was dispensed into the gasket slide and assembled to the LncRNA expression microarray slide. The slides were incubated for 17 hours at 65°C in an Agilent Hybridization Oven. The hybridized arrays were washed, fixed, and scanned using the Agilent DNA Microarray Scanner (part number G2505C). Quality control for labeling efficiency was shown in Supplementary Table 2.

(2) Data analysis is as follows: slides were scanned at 5 lm/pixel resolution using an Axon GenePix 4000B scanner (Molecular Devices Corporation) piloted by GenePix Pro 6.0 software (Axon). Scanned images (TIFF format) were then imported into NimbleScan software (version 2.5) for grid alignment and expression data analysis. Expression data were normalized through quantile normalization and the Robust Multichip Average (RMA) algorithm included in the NimbleScan software. The probe level files and mRNA level files were generated after normalization. All gene level files were imported into Agilent GeneSpring GX software (version 11.5.1) and normalized by the quantile method; then, Combat software was used to adjust the normalized intensity to remove batch effects. Hierarchical clustering was performed using Agilent GeneSpring GX software (version 11.5.1). The analysis was performed by KangChen Biotech., Shanghai, China. Agilent Feature Extraction software (version 11.0.1.1) was used to analyze acquired array images. We have four samples; three were control MSCs and one is TLR3-activated MSCs. LncRNAs and mRNAs that have flags in Present or Marginal (all targets value) in at least 2 out of the 3 control MSCs samples were chosen for further data analysis. Differentially expressed LncRNAs and mRNAs with statistical significance between the two groups were identified through *P* value/FDR filtering. Hierarchical clustering and combined analysis were performed using homemade scripts. For gene ontology analysis, Fisher's exact test was used to determine whether the overlap between the differentially expressed gene list and the GO annotation list was greater than that expected by chance. The *P* value denotes the significance of GO term enrichment in the differentially expressed genes.

## 3. Results

### 3.1. Characterization of AD-MSCs and the Expression Pattern of TLRs

**Figure 1:**
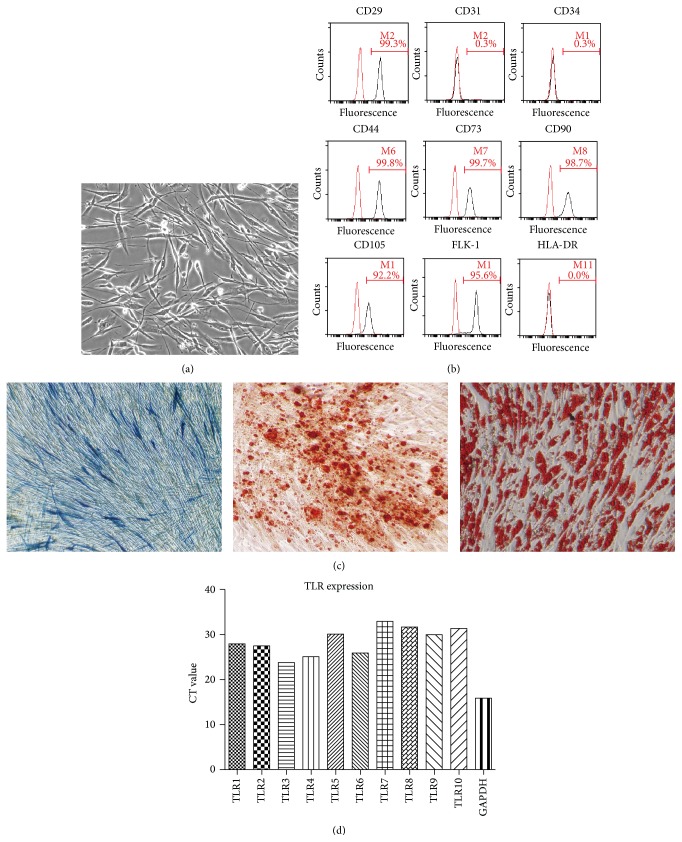
Characterization of AD-MSCs and the expression pattern of TLRs. (a) The cell morphology of AD-MSCs observed under light microscope. (b) Immunophenotype of AD-MSCs. (c) Differentiation capacity of AD-MSCs was demonstrated by ALP staining (left) and Alizarin red staining (centered) for osteoblasts and oil red O staining for adipocytes (right). (d) The expression levels of TLRs were analyzed by real-time PCR.

We isolated and expanded plastic adherent, spindle-like mesenchymal stem cells from adipose tissue (Figure 1(a)). AD-MSCs expressed high levels of CD29, CD44, and CD105 but were persistently negative for CD31, CD34, and HLA-DR (Figure 1(b)). Osteogenic differentiation was detected by Alizarin red staining and ALP activity assay (Figure 1(c)). Adipogenic differentiation was demonstrated by oil red O staining (Figure 1(c)). The expression of TLRs in MSCs remains controversial. PCR primers specific to TLR-1 and to TLR-10 were designed to detect TLRs from total RNA in AD-MSCs (Table 1). RT-PCR results showed that all TLRs were detectable within 33 cycles and that TLR3 has the highest expression level (Figure 1(d)).

### 3.2. Effect of TLR3 Agonist on Cytokine Expression of AD-MSCs

**Figure 2:**
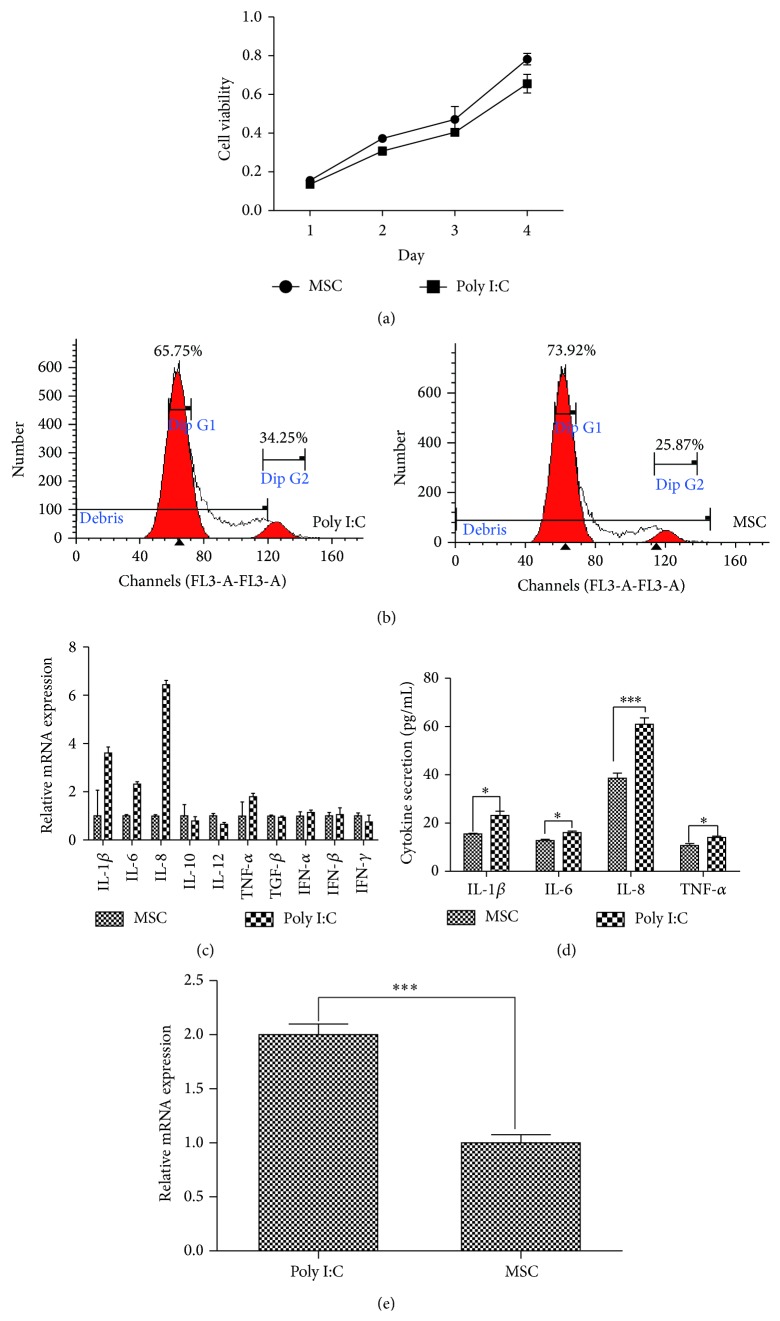
Effect of TLR3 agonist on AD-MSCs. (a) Effect of TLR3 agonist on the proliferation of AD-MSCs. (b) Cell cycle analysis of Poly I:C stimulated AD-MSCs and control AD-MSCs. (c) Cytokine secretion of AD-MSCs after Poly I:C stimulation as detected by RT-PCR. (d) Cytokine secretion of AD-MSCs after Poly I:C stimulation as detected by ELASA. (e) TLR3 expression in Poly I:C stimulated AD-MSCs and control AD-MSCs.

Since TLR3 had the highest expression level, to evaluate its functionality, we stimulated AD-MSCs with TLR3 agonist (Poly I:C 20 *μ*g/mL) for 24 h and determined its effect on cell proliferation and cytokine secretion. Poly I:C slightly decreased proliferation of AD-MSCs (Figure 2(a)). Flow cytometry analysis of cell cycles showed that Poly I:C stimulated AD-MSCs had enhanced G2 phase (Figure 2(b)). We measured a number of cytokines known to be involved in immunomodulation, including IL-1*β*, IL-4,IL-6, IL-8, IL-10, IL-12, TNF-*α*, TGF*β*, and IFN-*γ*. We found that Poly I:C induced upregulation of IL-1*β*, IL-6, IL-8, and TNF-*α* as measured by RT-PCR (Figure 2(c)). Enhanced release of these cytokines was also detected by ELISA (Figure 2(d)). Exposure to poly (I:C) also increased the expression of TLR3 (Figure 2(e)).

### 3.3. Overview of lncRNA and mRNA Profiles in TLR3-Activated AD-MSCs and Control AD-MSCs

**Figure 3:**
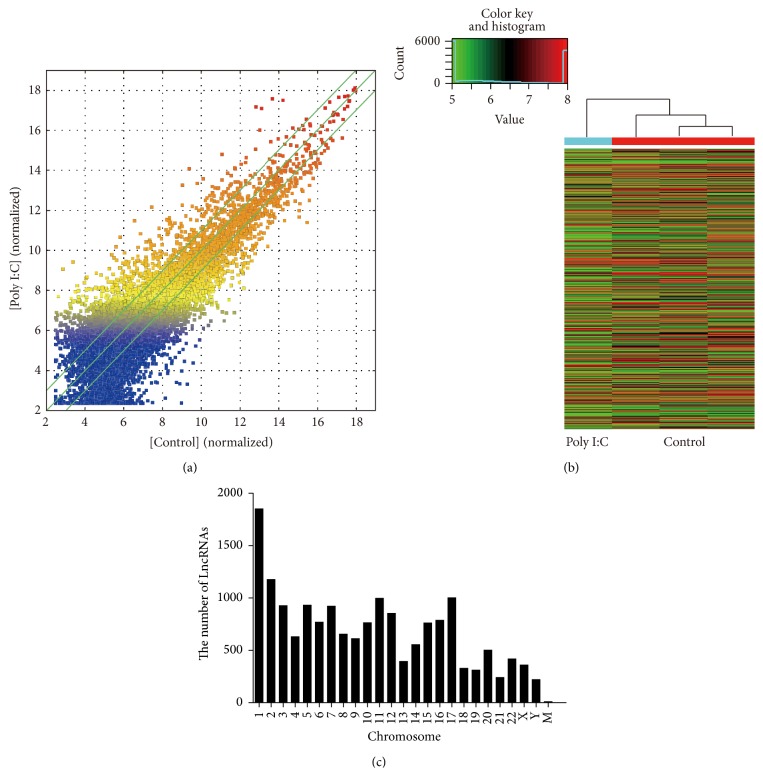
Overview of lncRNA profiles in TLR3-activated AD-MSCs and control AD-MSCs. (a) The scatter plot is a visualization method used for assessing the lncRNA expression variations between TLR3-activated AD-MSCs and control AD-MSCs. The values of the *X* and *Y* axes in the scatter plot are the averaged normalized signal values of the group (log 2 scale). The green lines are fold-change lines (the default fold-change given is 2). (b) Hierarchical clustering of lncRNAs in TLR3-activated AD-MSCs and control AD-MSCs. (c) Chromosomal distribution of detected lncRNAs.

To examine the lncRNA expression profiles in AD-MSCs treated with or without TLR3 agonist, we used Arraystar Human LncRNA Microarray V3.0 which contains 30,586 lncRNA probes, collected from RefSeq, UCSC knowngenes, Gencode, and so forth, and 26,109 mRNA probes. The overview of lncRNA expression profiles is summarized in Table 2 and Figure 3(a). Overall, we found that 55.5% of lncRNAs (16967 out of 30586) and 44.5% of protein-coding mRNA transcripts (11632 out of 26109) on the microarray exhibited expression above background. 10.2% of lncRNAs (1733 out of 16967) and 15.1% of protein-coding mRNA transcripts (1760 out of 11632) were significantly differentially expressed (absolute fold-change ≥5, *P* value ≤ 0.05) between TLR3-activated AD-MSCs and control AD-MSCs. The top 10 up- and downregulated LncRNAs in TLR3-activated AD-MSCs compared to control AD-MSCs were shown in Table 3.  Figure 3(b) showed the hierarchical cluster of lncRNAs expression in Poly I:C stimulated AD-MSCs and nonstimulated AD-MSCs. Additionally, statistical analysis showed that the expressed lncRNAs were widely distributed on all chromosomes (Figure 3(c)) and that chr1 had the most expressed lncRNAs, while the mitochondrial genome had the least.

### 3.4. Characteristics of LncRNAs with Changed Expression in TLR3-Activated AD-MSCs

**Figure 4:**
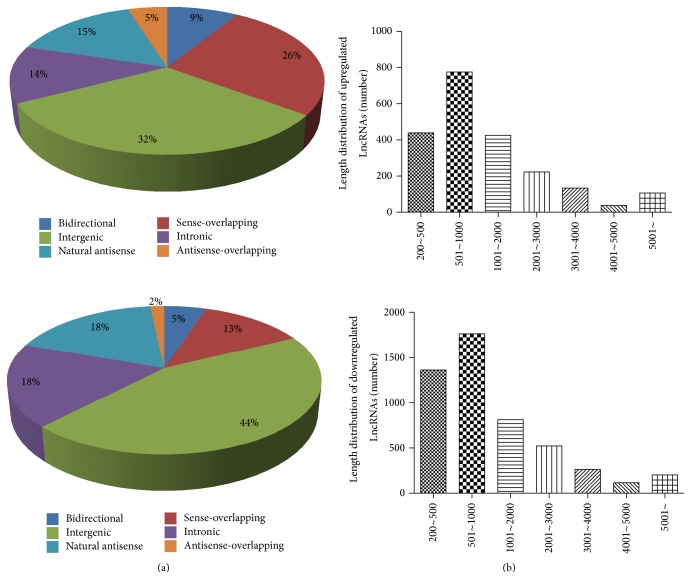
Characteristics of LncRNAs with changed expression in TLR3-activated AD-MSCs. (a) Distribution of various classes of differentially expressed LncRNAs. The ratio of 6 classes (sense overlap LncRNAs, antisense overlap LncRNAs, bidirectional LncRNAs, and intergenic LncRNAs) in total changed LncRNAs was analyzed in TLR3-activated AD-MSCs. (b) Length distribution of differentially expressed lncRNAs in TLR3-activated AD-MSCs.

Among the 7271 differentially expressed LncRNA (absolute fold-change ≥2, *P* value ≤ 0.05), 2155 lncRNAs were upregulated in experimental group compared to the control group, while 5116 lncRNAs were downregulated. Here, one upregulated lncRNA uc010kun.2 was of particular interest for us because it is located upstream of IL6, a cytokine induced after TLR3 activation in AD-MSCs. Using qPCR, we observed a 2.70-fold upregulation in uc010kun.2, consistent with microarray analysis results (2.63-fold-change) (Supplementary Figure 1). Table 3 showed the top 10 up- and downregulated lncRNAs in TLR3-activated AD-MSCs versus control AD-MSCs. We classified these differentially expressed LncRNAs into 6 groups: “sense-overlapping,” the LncRNA's exon is overlapping a coding transcript exon on the same genomic strand; “intronic,” the LncRNA is overlapping the intron of a coding transcript on the same genomic strand; “natural antisense,” the LncRNA is transcribed from the antisense strand and overlapping with a coding transcript; “nonoverlapping antisense,” the LncRNA is transcribed from the antisense strand without sharing overlapping exons; “bidirectional,” the LncRNA is oriented head to head to a coding transcript within 1000 bp; “intergenic”: there are no overlapping or bidirectional coding transcripts nearby the LncRNA.  Figure 4(a) showed the distribution of the six classes of LncRNAs with changed expression in TLR3-activated AD-MSCs. The lncRNAs are mainly between 200 bp and 3000 bp in length.  Figure 4(b) showed the length distribution of differentially expressed lncRNAs. The majority of the differentially expressed lncRNAs have a length between 500 bp and 1000 bp.

### 3.5. Gene Ontology and Pathway Analysis

**Figure 5:**
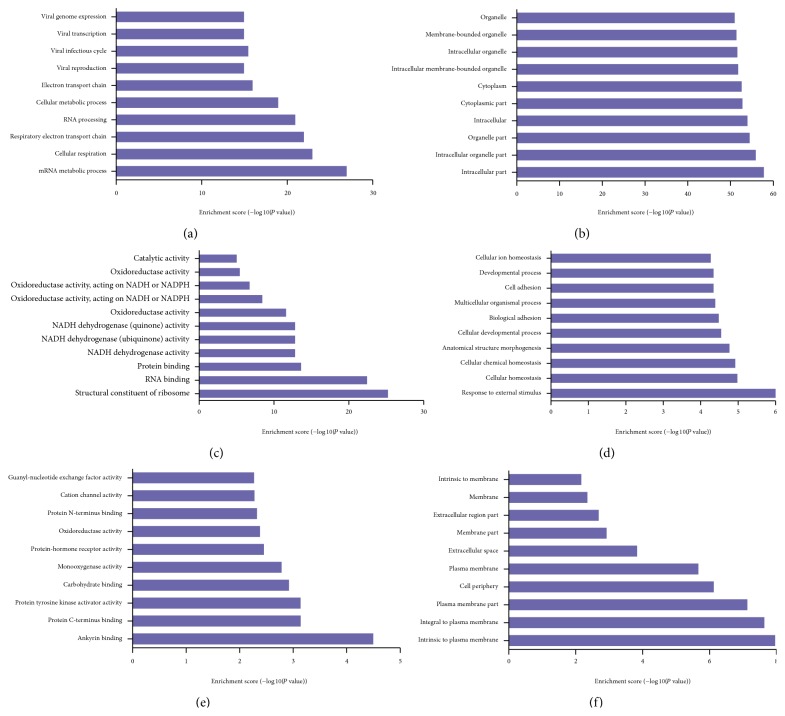
GO term enrichment in the differentially expressed genes. (a, b, c) Genes upregulated in TLR3-activated AD-MSCs. (d, e, f) Genes downregulated in TLR3-activated AD-MSCs.

**Figure 6:**
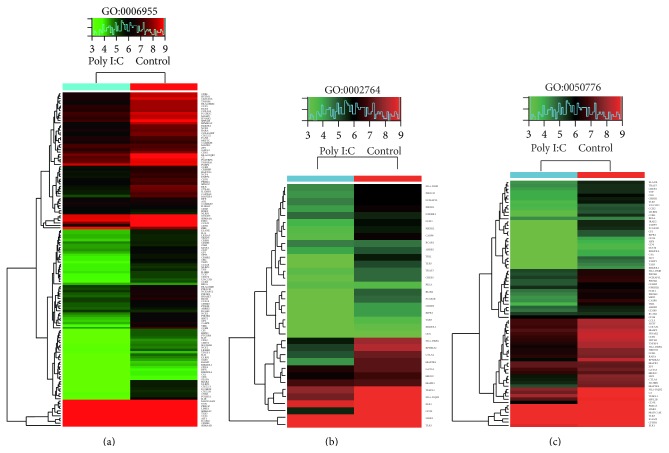
Gene ontology analysis of genes (a) associated with immune response GO:0006955, (b) genes associated with immune response-regulation signaling pathway GO:0002764, and (c) genes associated with regulation of immune response GO:0050776.

**Figure 7:**
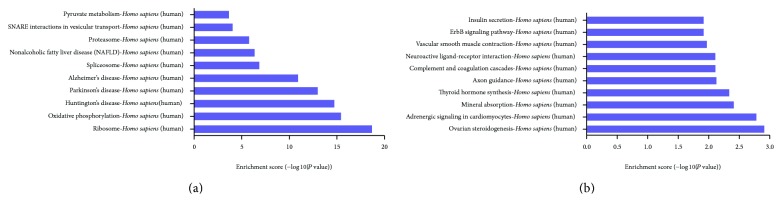
Pathway analysis of the differentially expressed genes. (a) Top 10 of the upregulated pathways in TLR3-activated AD-MSCs. (b) Top 10 of the downregulated pathways in TLR3-activated AD-MSCs.

GO analysis was performed to determine the gene and gene product enrichment in biological processes, cellular components, and molecular functions (Supplementary Figure 2). We found that the highest enriched GOs targeted by upregulated mRNAs in TLR3-activated MSCs were mRNA metabolic process (ontology: biological process) (Figure 5(a)), intracellular part (ontology: cellular component) (Figure 5(b)), and structural constituent of ribosome (ontology: molecular function) (Figure 5(c)). The highest enriched GOs targeted by the downregulated transcripts in TLR3-activated MSCs were response to external stimulus (ontology: biological process) (Figure 5(d)), ankyrin binding (ontology: cellular component) (Figure 5(e)), and intrinsic to plasma membrane (ontology: molecular function) (Figure 5(f)). Specifically, we analyzed expression of genes associated with immune response (GO:0006955 Figure 6(a)), genes associated with immune response-regulation signaling pathway (GO:0002764 Figure 6(b)), and genes associated with regulation of immune response (GO:0050776 Figure 6(c)). Additionally, other genes associated with immune response (GO:0050778, GO:0042092, GO:0002828, GO:0002920, and GO:0002821) were analyzed (Supplementary Figure 3). Pathway analysis indicated that 38 pathways were upregulated and 31 were downregulated in TLR3-activated MSCs.  Figure 7 showed the top 10 of the changed pathways.

## 4. Discussion

TLRs play an important role in innate and adaptive immunity. The expression and function of multiple TLRs have been described in many cell types, especially in cells of the innate immune system, where they function as sensors of infection or damage [8, 9]. However, conflicting results have been reported because TLRs expressed on different cell types from different species and tissues resulted in different responses [10]. Recently, it has been reported that MSC derived from adult bone marrow also expresses functional TLRs that promote their survival and proinflammatory cytokine secretion [11]. Here, we show that AD-MSCs express 10 types of TLRs and the expression level of TLR3 was the highest. Activation of TLR3 in AD-MSCs leads to enhanced secretion of IL-1*β*, IL-6, IL-8, and TNF-*α*.

It is well known that TLRs and their specific stimulus activate MyD88-dependent or -independent downstream signaling pathways. However, whether epigenetic regulators such as long noncoding RNA are involved in this process remains unclear. lncRNAs are over 200 nucleotides in length and are separate from the other known ncRNA subsets. Previous studies have focused primarily on short noncoding RNAs, such as microRNAs, transfer RNAs, and short interfering RNAs. Increasing evidence confirmed lncRNAs to be one of the most important factors controlling gene expression and the aberrant expression of which is involved in a variety of human diseases, including immunological disorders [12, 13]. Guttman et al. were the first to use the intergenic deposition of epigenetic marks to identify 20 lncRNAs induced in lipopolysaccharide- (LPS-) stimulated mouse bone marrow-derived dendritic cells (BMDD) [14]. Later on, several lncRNAs have been identified following activation of monocytes, macrophages, fibroblasts, and dendritic cells [15–18]. The potential importance of lncRNAs in the immune response is only now emerging. Immune-related lncRNAs are generally identified through examination of differential expression in response to activation of immune cells [6]. Here, a major focus of our study was to define the repertoire of lncRNAs in TLR3-activated AD-MSCs. We performed comprehensive lncRNA and mRNA profiling through microarray and found 10.2% of lncRNAs (1733 out of 16967) and 15.1% of mRNA transcripts (1760 out of 11632) were significantly differentially expressed in TLR3 stimulated MSCs. Furthermore, we characterized the differentially expressed lncRNAs through their classes and length distribution and correlated them with differentially expressed mRNA.

An interesting observation from sequencing data is that many of the immune-related lncRNAs are located close to, or partially overlapping, the 5′ end (upstream) or 3′ end (downstream) of protein-coding genes implicated in the immune response [6, 19]. Here, one upregulated lncRNA uc010kun.2 was of particular interest for us because it is located upstream of IL6, a cytokine induced after TLR3 activation in AD-MSCs. Another lncRNA our data may highlight is lncRNA-Cox7A2. Carpenter et al. indicated that Cox2 and lncRNA-Cox2 regulated by MyD88 and NF-kB were markedly induced after TLR4 stimulation in BMDMs [17]. Here, lncRNA-Cox7A2 is predicted to be associated with Cox7A2, which is also a cytochrome c oxidase subunit.

TLR3 signaling and subsequent inflammatory responses are controlled by a multitude of regulatory molecules. Here, we propose a model whereby TLR3 signaling induces expression changes of lncRNAs which then exert their effects as repressors or activators of genes through interactions with various regulatory complexes. As such, lncRNAs represent a new component of the TLR3 signaling pathway.

In summary, our study was the first to demonstrate that a set of lncRNAs is significantly regulated in AD-MSCs upon stimulation with TLR3, suggesting a role of lncRNAs in the immune response of AD-MSCs. This will provide the basis for the subsequent functional and mechanistic analysis of individual lncRNAs. Currently, the precise functions of the differentially expressed lncRNAs remain largely unknown and advances in the understanding of their role will allow us to tackle a range of challenges in AD-MSCs immunomodulatory properties.

## Highlights

1. TLR3 is highly expressed in adipose tissue derived mesenchymal stem cells.
2. TLR3 agonist Poly I:C promotes secretion of a variety of cytokines.
3. TLR3-activated AD-MSCs show dynamic changes in lncRNA and mRNA profiles.

## Supplementary Material

Supplementary Table 1 demonstrated the RNA quantification and quality assurance of four samples (one is TLR3-activated MSCs, three are control MSCs); table 2 showed the quality control for labeling efficiency during the microarray. Supplementary figure 1 showed the consistence between microarray and qPCR results using uc010kun.2 as an example; figure 2 was GO analysis of differentially expressed genes according to biological process, cellular component and molecular function classification; figure 3 was GO analysis of a set of specific genes associated with immune response.

## Figures and Tables

**Table 1:** PCR primers specific to TLR-1 and to TLR-10.

Gene	Sense primer (5′~3′)	Antisense primer (5′~3′)
TLR1	CCACGTTCCTAAAGACCTATCCC	CCAAGTGCTTGAGGTTCACAG
TLR2	ATCCTCCAATCAGGCTTCTCT	GGACAGGTCAAGGCTTTTTACA
TLR3	TTGCCTTGTATCTACTTTTGGGG	TCAACACTGTTATGTTTGTGGGT
TLR4	AGACCTGTCCCTGAACCCTAT	CGATGGACTTCTAAACCAGCCA
TLR5	TCCCTGAACTCACGAGTCTTT	GGTTGTCAAGTCCGTAAAATGC
TLR6	TGAATGCAAAAACCCTTCACCT	CCAAGTCGTTTCTATGTGGTTGA
TLR7	CACATACCAGACATCTCCCCA	CCCAGTGGAATAGGTACACAGTT
TLR8	ATGTTCCTTCAGTCGTCAATGC	TTGCTGCACTCTGCAATAACT
TLR9	CTGCCACATGACCATCGAG	GGACAGGGATATGAGGGATTTGG
TLR10	GGTTCTTTTGCGTGATGGAATC	GGTCGTCCCAGAGTAAATCAAC

**Table 2:** Summary of microarray analysis results.

Probe class	Total	Expressed above background	Differentially expressed^*∗*^
LncRNA mRNA Combined	30586 26109 56695	16967 (55.5%) 11632 (44.5%) 28599 (50.4%)	1733 (10.2%) 1760 (15.1%) 3493 (12.2%)

^*∗*^Significant differential expression was defined as probes with *P*≤ 0.05 and absolute fold-change ≥5.

**Table 3:** The top 10 up- and downregulated lncRNAs in TLR3-activated AD-MSCs versus control AD-MSCs.

	Top 10 LncRNAs	Chromosomal localization	RNA length	Start locus	Stop locus	Associated gene name	Relationship
TLR3-activated AD-MSCs versus control AD-MSCs upregulated	NR_052024	Chr20	803	33866708	33872520	EIF6	Sense-overlapping
	NR_037793	Chr14	1853	50065414	50081390	LRR1	Sense-overlapping
	ENST00000577672	Chr1	409	1655950	1667412	SLC35E2	Intronic
	ENST00000430598	Chr15	4279	20613648	20711433		Intergenic
	ENST00000514727	Chr4	442	140036086	140036528		Intergenic
	NR_024596	Cchr11	1129	86014397	86056985	C11orf73	Sense-overlapping
	NR_027653	Chr10	4525	3818187	3827473	KLF6	Sense-overlapping
	uc002xij.3	Chr20	805	37049238	37063962		Intergenic
	ENST00000478666	Chr3	1068	75471569	75484197		Intergenic
	uc010hbj.3	Chr22	1172	51222224	51238065	RABL2B	Bidirectional

TLR3-activated AD-MSCs versus control AD-MSCs downregulated	TCONS_00020653	Chr12	978	130442131	130444607		Intergenic
	ENST00000428453	Chr15	4383	20588367	20711414		Intergenic
	CB112975	Chr13	379	30229248	30229615		Intergenic
	uc009whu.1	Chr1	2772	142853227	142855999		Intergenic
	TCONS_00013	Chr7	1092	22450335	22452159		Intergenic
	ENST00000557155	Chr14	381	85860291	85886396		Intergenic
	ENST00000421735	Chr3	338	50304072	50304803	SEMA3B	Bidirectional
	TCONS_00014720	Chr8	206	59129760	59131635		Intergenic
	ENST00000505736	Chr4	1668	137717876	138133953		Intergenic
	ENST00000568150	Chr16	547	48657346	48778553		Intergenic

## References

[1] Lin R., Wang S., Zhao R. C. (2013). Exosomes from human adipose-derived mesenchymal stem cells promote migration through Wnt signaling pathway in a breast cancer cell model. *Molecular and Cellular Biochemistry*.

[2] Pevsner-Fischer M., Morad V., Cohen-Sfady M. (2007). Toll-like receptors and their ligands control mesenchymal stem cell functions. *Blood*.

[3] Beutler B. (2004). Inferences, questions and possibilities in Toll-like receptor signalling. *Nature*.

[4] De Luca K., Frances-Duvert V., Asensio M.-J. (2009). The TLR1/2 agonist PAM_3_CSK_4_ instructs commitment of human hematopoietic stem cells to a myeloid cell fate. *Leukemia*.

[5] Qi C., Xiaofeng X., Xiaoguang W. (2014). Effects of toll-like receptors 3 and 4 in the osteogenesis of stem cells. *Stem Cells International*.

[6] Heward J. A., Lindsay M. A. (2014). Long non-coding RNAs in the regulation of the immune response. *Trends in Immunology*.

[7] Cao Y., Sun Z., Liao L., Meng Y., Han Q., Zhao R. C. (2005). Human adipose tissue-derived stem cells differentiate into endothelial cells in vitro and improve postnatal neovascularization in vivo. *Biochemical and Biophysical Research Communications*.

[8] Kulkarni R., Behboudi S., Sharif S. (2011). Insights into the role of Toll-like receptors in modulation of T cell responses. *Cell and Tissue Research*.

[9] Smith P. D., Shimamura M., Musgrove L. C. (2014). Cytomegalovirus enhances macrophage TLR expression and MyD88-mediated signal transduction to potentiate inducible inflammatory responses. *The Journal of Immunology*.

[10] DelaRosa O., Lombardo E. (2010). Modulation of adult mesenchymal stem cells activity by toll-like receptors: implications on therapeutic potential. *Mediators of Inflammation*.

[11] Raicevic G., Najar M., Stamatopoulos B. (2011). The source of human mesenchymal stromal cells influences their TLR profile as well as their functional properties. *Cellular Immunology*.

[12] Chen L.-L., Carmichael G. G. (2010). Decoding the function of nuclear long non-coding RNAs. *Current Opinion in Cell Biology*.

[13] Turner M., Galloway A., Vigorito E. (2014). Noncoding RNA and its associated proteins as regulatory elements of the immune system. *Nature Immunology*.

[14] Guttman M., Amit I., Garber M. (2009). Chromatin signature reveals over a thousand highly conserved large non-coding RNAs in mammals. *Nature*.

[15] Rapicavoli N. A., Qu K., Zhang J., Mikhail M., Laberge R.-M., Chang H. Y. (2013). A mammalian pseudogene lncRNA at the interface of inflammation and antiinflammatory therapeutics. *eLife*.

[16] Wang P., Xue Y., Han Y. (2014). The STAT3-binding long noncoding RNA lnc-DC controls human dendritic cell differentiation. *Science*.

[17] Carpenter S., Aiello D., Atianand M. K. (2013). A long noncoding RNA mediates both activation and repression of immune response genes. *Science*.

[18] Dave R. K., Dinger M. E., Andrew M., Askarian-Amiri M., Hume D. A., Kellie S. (2013). Regulated expression of *PTPRJ*/CD148 and an antisense long noncoding RNA in macrophages by proinflammatory stimuli. *PLoS ONE*.

[19] Hu G., Tang Q., Sharma S. (2013). Expression and regulation of intergenic long noncoding RNAs during T cell development and differentiation. *Nature Immunology*.

