# Supplementary material for: Transcriptome Analysis of Long Noncoding RNAs in Toll-Like Receptor 3-Activated Mesenchymal Stem Cells
Source: Stem Cells Int. 2015 Nov 23;2016:6205485. doi: 10.1155/2016/6205485 (PMC4670881; doi:10.1155/2016/6205485)

**Supplementary information**

Supplementary Table 1 RNA Quantification and Quality Assurance by NanoDrop ND-1000

| \| Sample ID \| \| --- \| | \| OD260/280 Ratio \| \| --- \| | \| OD260/230 Ratio \| \| --- \| | \| QC* result Pass or Fail \| \| --- \| |
| --- | --- | --- | --- | --- | --- | --- | --- |
| Control 1 | 2.00 | 2.74 | Pass |
| Control 2 | 2.04 | 2.70 | Pass |
| Control 3 | 1.87 | 2.03 | Pass |
| Poly I:C | 2.04 | 2.29 | Pass |

*For spectrophotometer, the O.D. A260 /A280 ratio should be close to 2.0 for pure RNA (ratios between 1.8 and 2.1 are acceptable). The O.D. A260/A230 ratio should be more than 1.8.

Supplementary Table 2 Quality control for labeling efficiency

| \| Sample ID \| \| --- \| | \| Dye Name \| \| --- \| | \| Specific activity* \| \| --- \| | \| QC* result Pass or Fail \| \| --- \| |
| --- | --- | --- | --- | --- | --- | --- | --- |
| Control 1 | Cy3 | 28.01 | Pass |
| Control 2 | Cy3 | 26.00 | Pass |
| Control 3 | Cy3 | 23.15 | Pass |
| Poly I:C | Cy3 | 22.98 | Pass |

*The specific activity (pmol dyes per μg cRNA) of the labeled RNA can be obtained by the following calculation: Specific Activity =(pmol per μl dye) / (μg per μl cRNA). For two-color, the specific activity should>8.0 pmol Cy3 or Cy5, for one-color, the specific activity should >9.0 pmol Cy3 or Cy5.

Supplementary Figure 1 Comparison between microarray data and qPCR results for LncRNA uc010kun.2

Supplementary Figure2 Gene ontology analysis of the differentially expressed genes according to biological process, cellular component and molecular function classification.(A,B,C) genes up-regulated in TLR3-activated AD-MSCs. (D,E,F) genes down-regulated in TLR3-activated AD-MSCs.

Supplementary Figure3 Gene ontology analysis of (A) genes associated with positive regulation of adaptive immune response (GO:0002821) or (B) genes associated with regulation of type 2 immune response (GO:0002828)or (C) genes associated with regulation of humoral immune response(GO:0002920) or (D) genes associated with type 2 immune response (GO:0042092) or (E) genes associated with positive regulation of immune response(GO:0050778).

Supplementary Figure 1


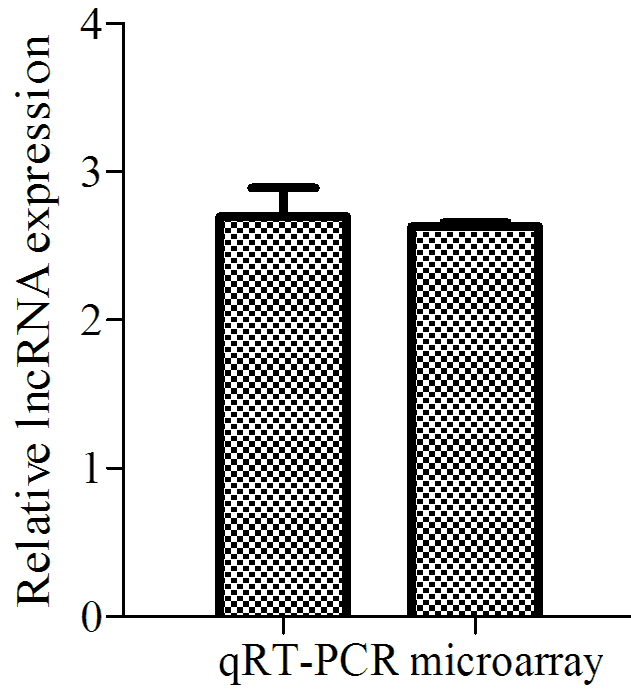


Supplementary Figure 2


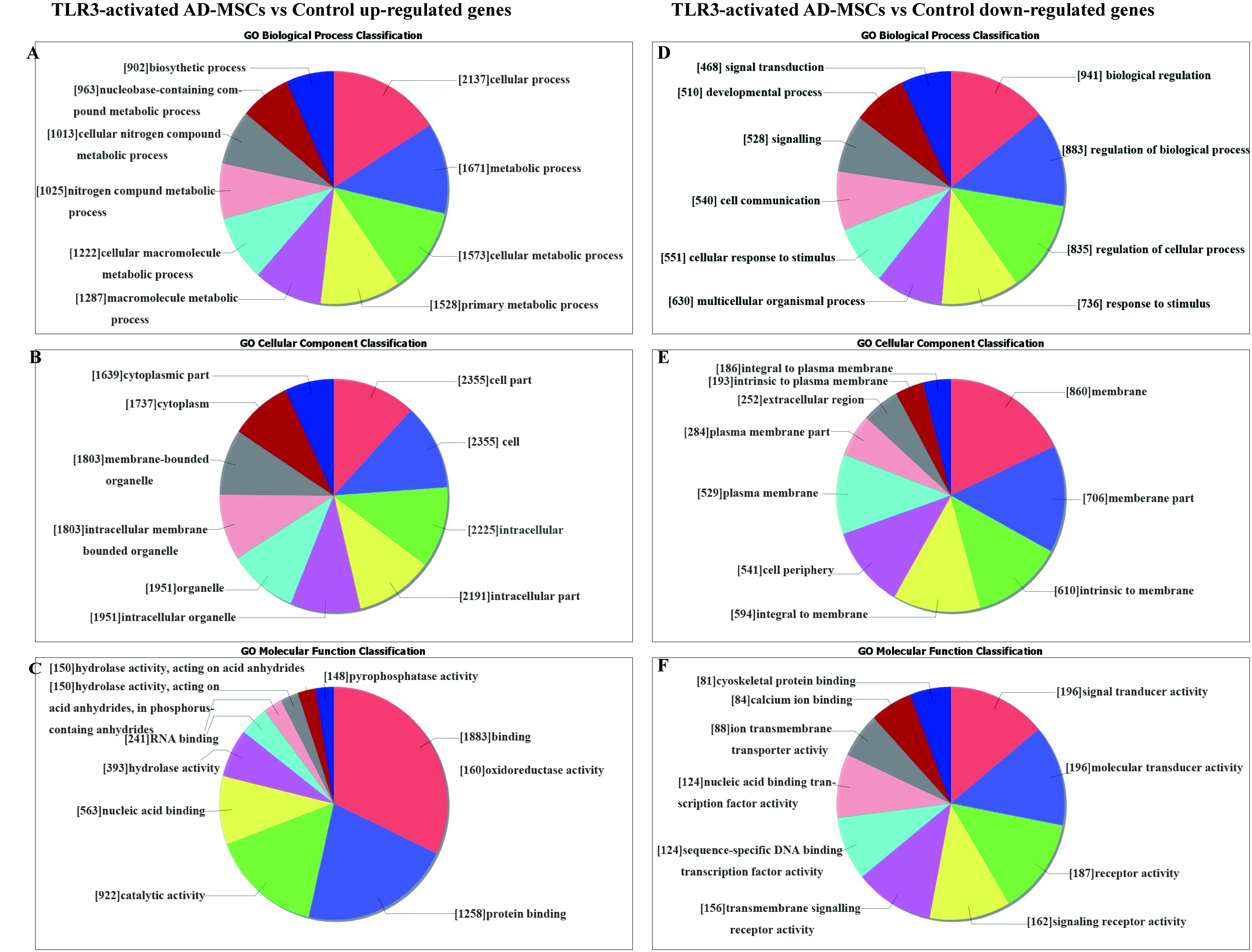


Supplementary Figure3


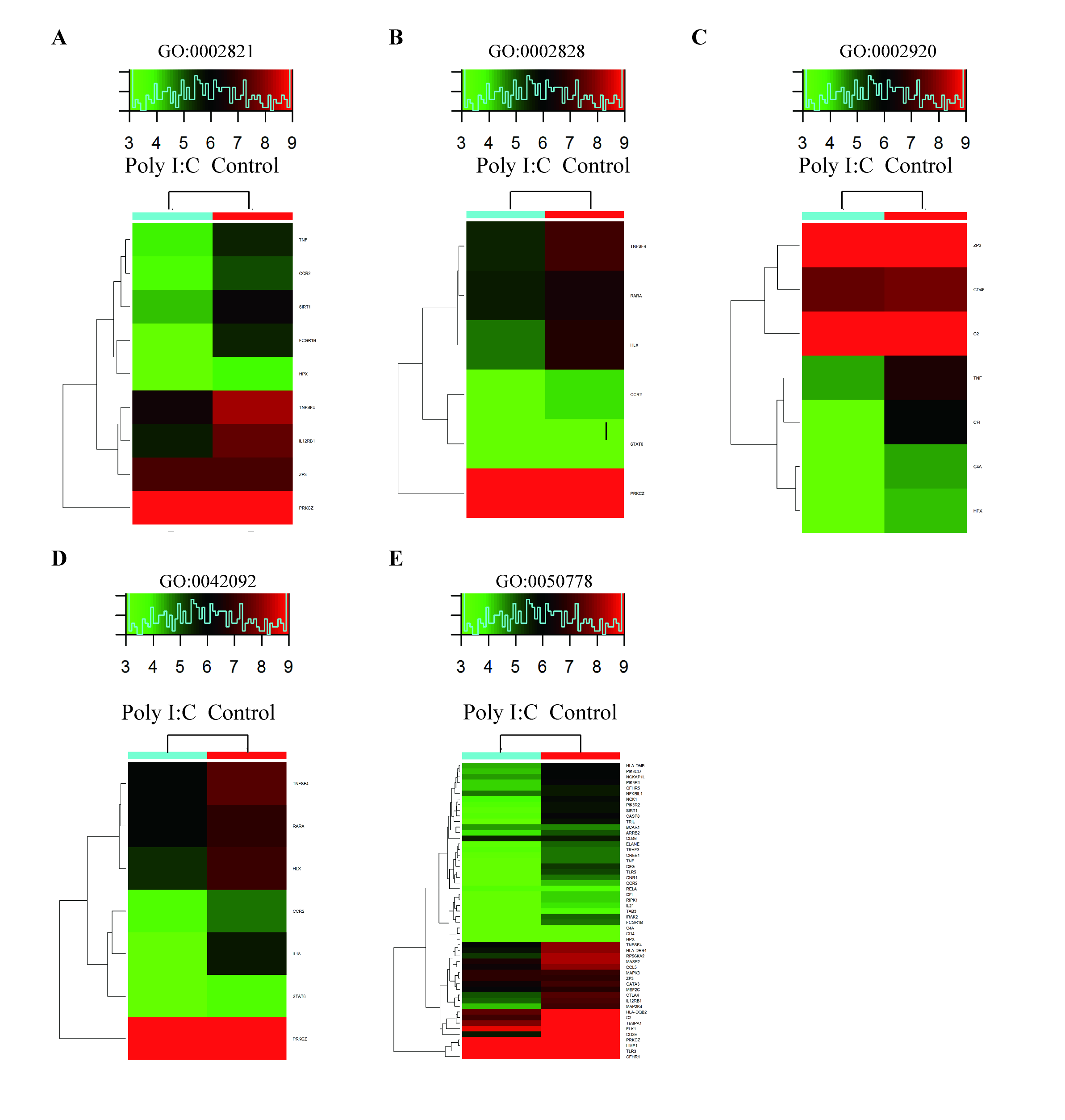

Supplement: Supplementary file 1 — Supplementary Table 1 demonstrated the RNA quantification and quality assurance of four samples (one is TLR3-activated MSCs, three are control MSCs); table 2 showed the quality control for labeling efficiency during the microarray. Supplementary figure 1 showed the consistence between microarray and qPCR results using uc010kun.2 as an example; figure 2 was GO analysis of differentially expressed genes according to biological process, cellular component and molecular function classification; figure 3 was GO analysis of a set of specific genes associated with immune response. [file 6205485.f1.docx]
